# Supplementary material for: Optoribogenetic control of regulatory RNA molecules
Source: Nat Commun. 2020 Sep 24;11:4825. doi: 10.1038/s41467-020-18673-5 (PMC7518282; doi:10.1038/s41467-020-18673-5)
Supplement: Supplementary file 1 — Supplementary Information [file 41467_2020_18673_MOESM1_ESM.pdf]

Supplementary Information

Optoribogenetic control of regulatory RNA molecules

Sebastian Pilsl[1], Charles Morgan[1], Moujab Choukeife[1], Andreas Möglich[2], and Günter Mayer[1,3]*

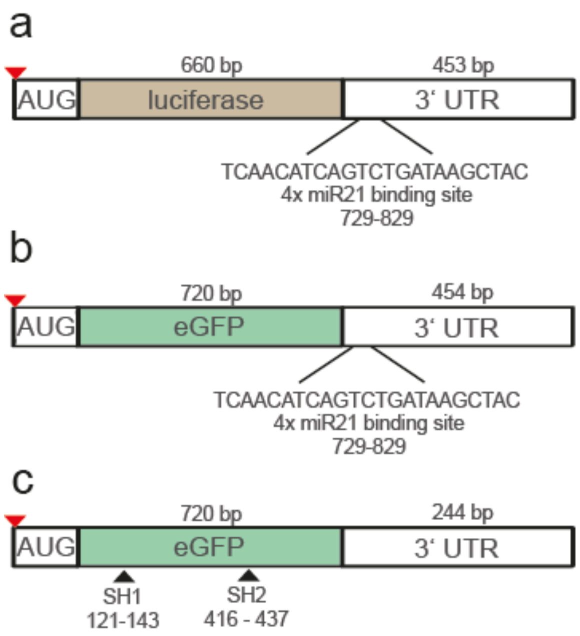

Supplementary Figure 1: Schematics of regulatory RNA binding sites on luciferase and eGFP reporter mRNA molecules. Schematic representations of luciferase (**a**) and eGFP mRNA (**b**) coding regions (brown (**a**), green(**b**)) and corresponding 3' untranslated regions (3'UTRs) with four miR21 binding sites were used as reporter mRNAs for *pre*-miR21 experiments (**Fig. 2**). **c**, Schematic representation of eGFP reporter mRNA for shRNA experiments (**Fig. 3**) with no miR21 binding sites embedded in the 3'UTR. **c**, Putative binding sites of siRNAs originating from SH1 and SH2 are indicated by black arrows. **a-c**, Numbers indicate base numbers relative to translation to start codon as well as the length of protein coding regions and the 3'UTR regions.

5’GCGGCCGCTCAACATCAGTCTGATAAGCTACTAATCAACATCAGTCTGATAAGCTACTAATCAA
CATCAGTCTGATAAGCTACTAATCAACATCAGTCTGATAAGCTAGCGGCCGCGACTCTAGATCAT
AATCAGCCATACCACATTTGTAGAGGTTTTACTTGCTTTAAAAAACCTCCCACACCTCCCCCTGAA
CCTGAAACATAAAATGAATGCAATTGACAGCCCATCGACTGGTGTTGCTAAACAGCCCATCGACT
GGTGTTGCTAAACAGCCCATCGACTGGTGTTGCTAAACAGCCCATCGACTGGTGTTGCAATTG3’

Supplementary Figure 2: DNA sequence of the miR21 binding sequences located in the 3’UTR of the reporter plasmids. Four binding sites (blue) complementary to miR21-5p were cloned into the NotI restriction site (grey). Four binding sites (green) complementary to miR21-3p were cloned into the MfeI restriction site (grey).

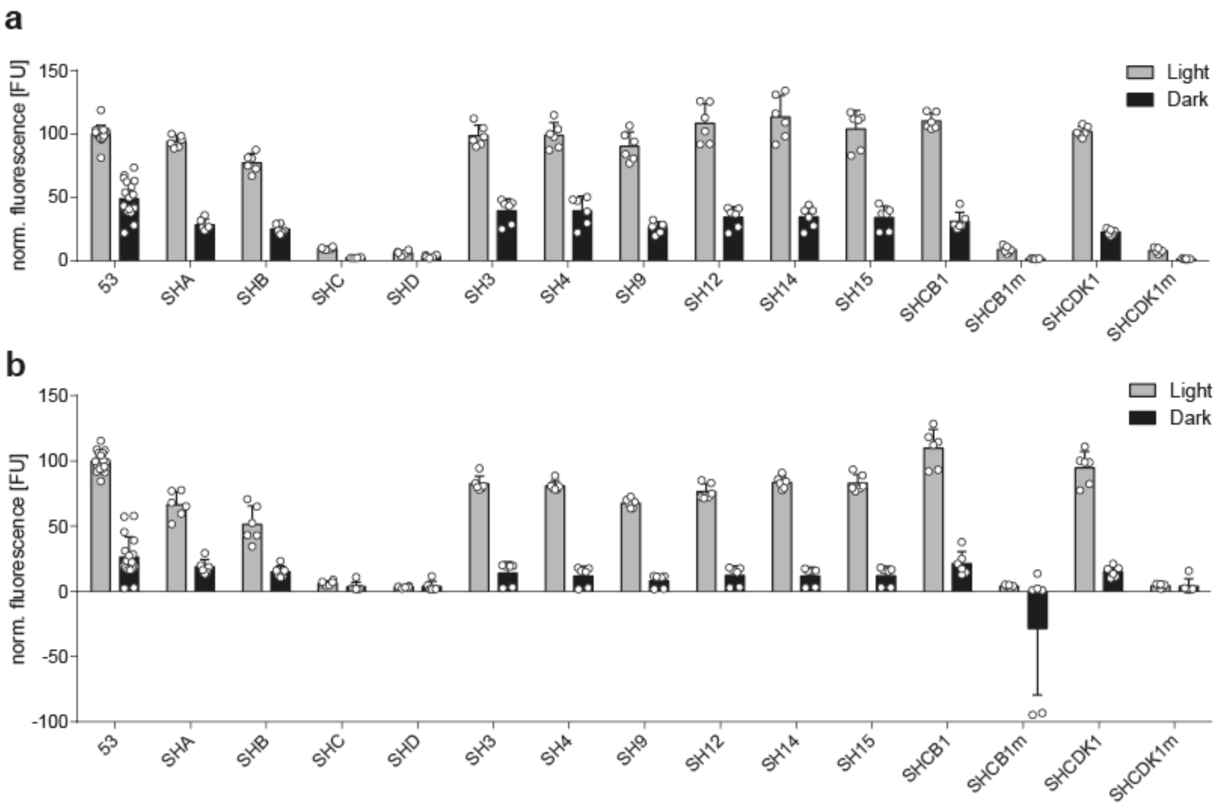

Supplementary Figure 3: Aptamer-conjoined regulatory RNAs bind to PAL light-dependently *in vitro*, whereas binding is reduced for respective point mutants. Biotinylated PAL protein was immobilized on streptavidin coated wells. Binding of 1000 nM (**a**) or 100 nM (**b**) *pre*-miR21, eGFP shRNA or cell cycle regulatory RNA constructs was quantified in presence of 0.5 mg mL^{-1} heparin and 0.5 mg mL^{-1} BSA by RiboGreen fluorescence. **a**,**b**, Values are processed by subtracting background fluorescence from equally treated wells without immobilized PAL and subsequent normalization to 53 incubated under light conditions. **a**,**b**, N = 3. Each biologically independent experiment was performed in duplicates. Grey bars: light conditions, black bars: darkness. Values are means ± s. d. Source data are provided as a source data file.

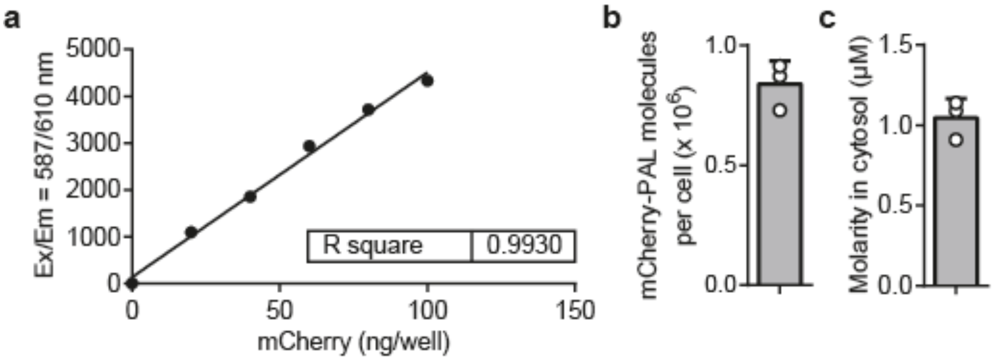

Supplementary Figure 4: Quantification of mCherry-PAL expression levels in the transgenic cell line HEK293PAL. a, Representative standard curve for determination of the linear range of mCherry quantification. **b**, Determination of average mCherry-PAL molecules per cell using the corresponding mCherry standard curve. **c**, mCherry-PAL molarity in the cytosol calculated from (**b**). N = three biologically independent experiments performed once. Values are means ± s. d. Source data are provided as a source data file.

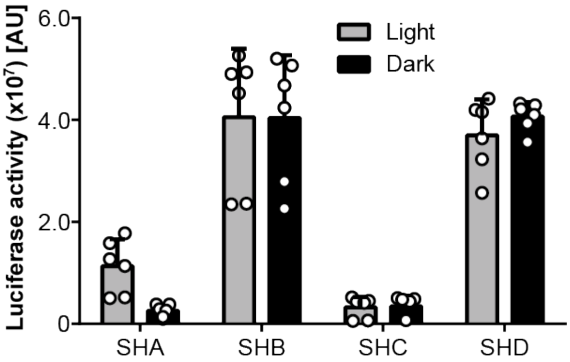

Supplementary Figure 5: A *pre*-miR21-aptamer chimera enables light-control of luciferase activity. Luciferase activity after transient transfection of the indicated *pre*-miR21 variants. HEK293PAL cells were incubated under the indicated light conditions prior to measurement. A normalized variant of this dataset is shown in **Fig. 2b.** N = 6. Each biologically independent experiment was performed in duplicates. Grey bars: light conditions, black bars: dark conditions. Values are means ± s.d. Source data are provided as a source data file.

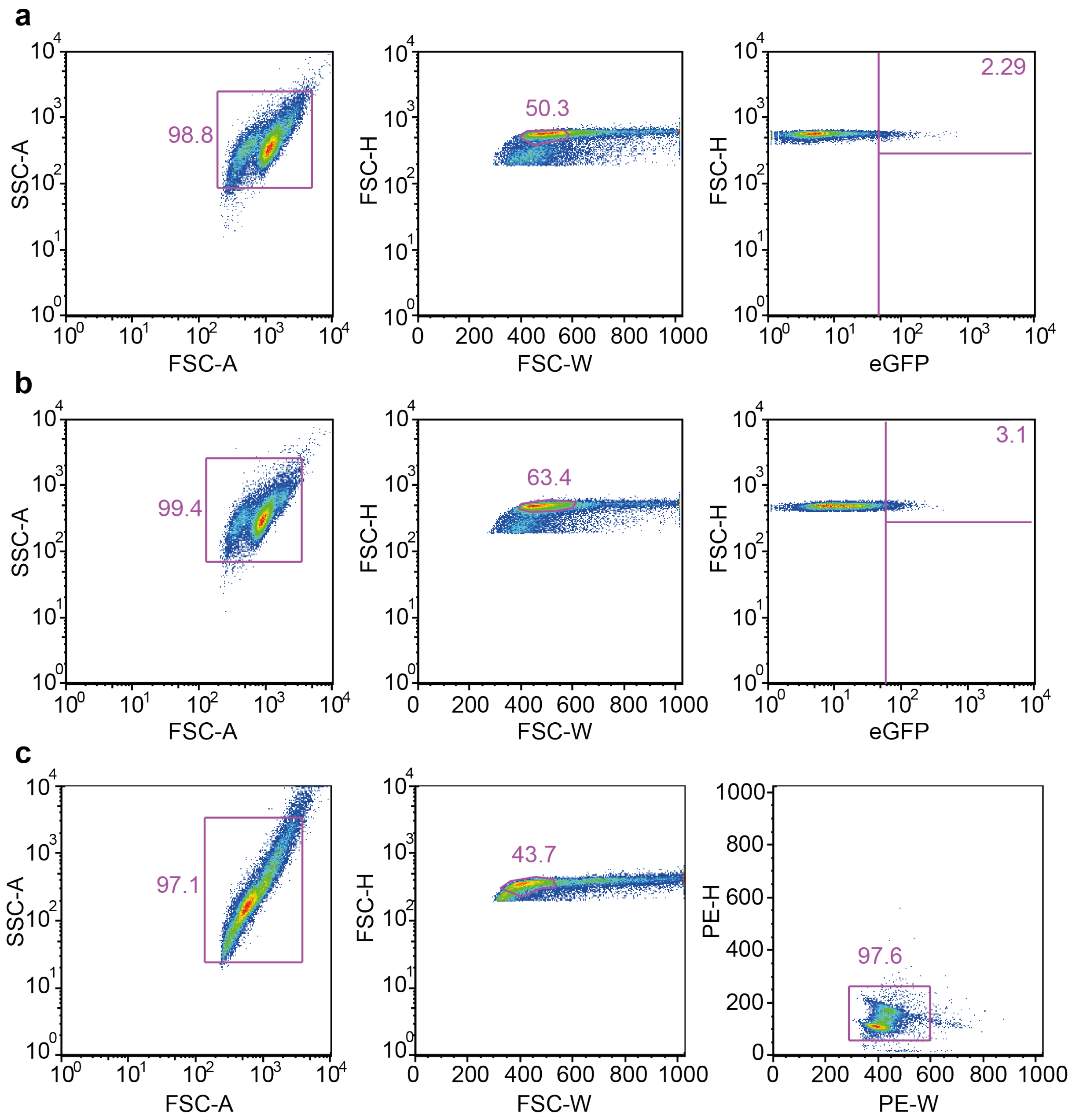

Supplementary Figure 6: Gating strategies to identify eGFP positive cells for miR21 (a), shRNA experiments (b) and cell cycle phases (c). a-c, Cell debris was excluded using side scatter area (SSC-A) *vs*. forward scatter area (FSC-A). Singlet cells were detected using forward scatter height (FSC-H) *vs*. forward scatter width (FSC-W). For miR21 and shRNA experiments (**a**, **b**), eGFP positive cells were identified using FSC-H *vs*. Fluorescein isothiocyanate area (depicted as eGFP). **a**, Cells transfected with SHA and incubated in darkness were set to 2.29 % eGFP positive cells and gating was applied to all other tested samples. **b**, Cells transfected with SH1 and incubated in darkness were set to 3.1 % eGFP positive cells and gating was applied to all other tested samples. **c**, Cell cycle debris and apoptotic cells were excluded using Phycoerythrin height (PE-H) *vs*. Phycoerythrin width (PE-W). Flow cytometry was performed using a BD FACS Canto II instrument (BD Bioscience). Data processing was performed using FlowJo (9.6.3) and GraphPad Prism (6.01).

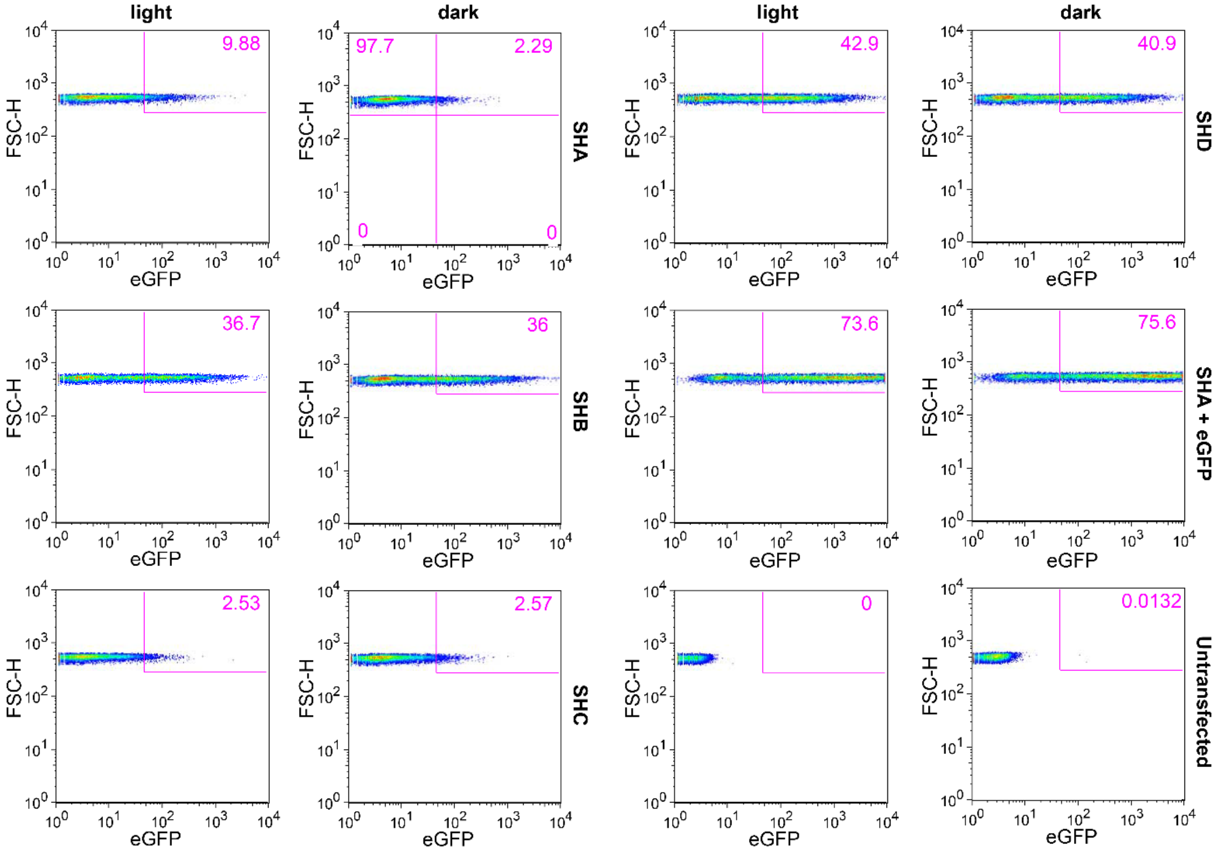

Supplementary Figure 7: Detection of eGFP expression for miR21 experiments in absence of AGO2 overexpression shown in Fig. 2d. Representative flow cytograms of HEK293PAL cells after transfection of the indicated *pre*-miR variants. Gating strategy was applied as outlined in **Supplementary Figure 6a**. HEK293PAL cells were incubated under the indicated light conditions prior to measurement. eGFP expression was detected by using forward scatter height (FSC-H) *vs*. Fluorescein isothiocyanate area (FITC-A) channel (eGFP). Flow cytometry was performed using a BD FACS Canto II instrument (BD Bioscience). Data processing was performed using FlowJo (9.6.3) and GraphPad Prism (6.01).

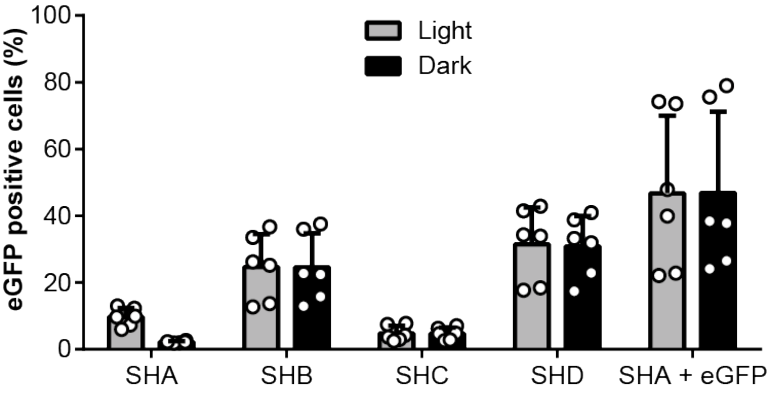

Supplementary Figure 8: A *pre*-miR21-aptamer chimera enables light-control of eGFP expression. Number of eGFP positive cells after transient transfection of the indicated *pre*-miR21 variants. HEK293PAL cells were incubated under the indicated light conditions prior to measurement. A normalized variant of this dataset is shown in **Fig. 2d.** eGFP expression was identified as indicated in **Supplementary Fig. 6a**. N = 6. Each biologically independent experiment was performed in duplicates. Grey bars: light conditions, black bars: dark conditions. Values are means ± s.d. Source data are provided as a source data file.

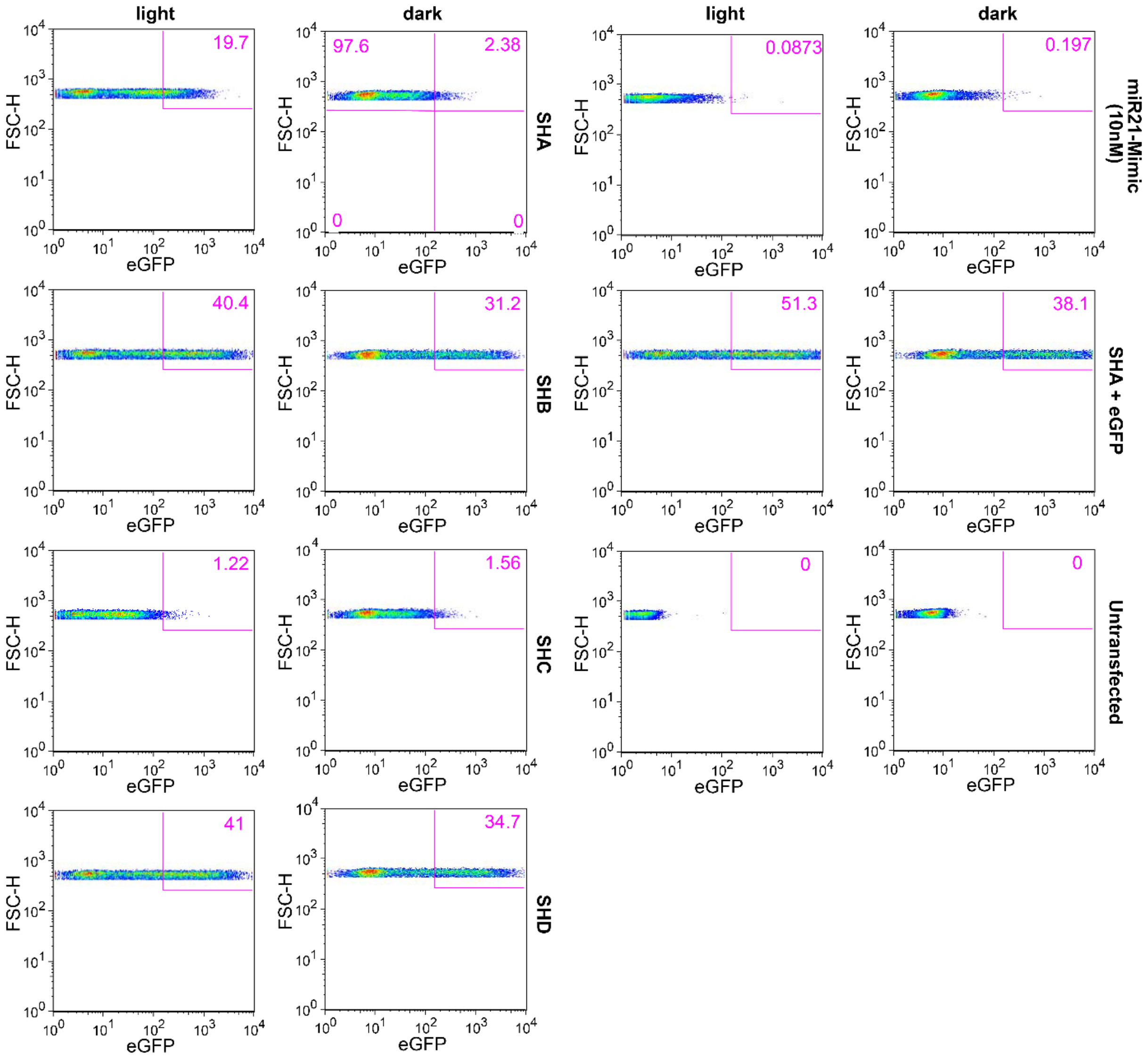

Supplementary Figure 9: Detection of eGFP expression for miR21 experiments in presence of elevated levels of AGO2 shown in Fig. 2f. Representative flow cytograms of HEK293PAL cells after transfection of the indicated *pre*-miR variants. Gating strategy was applied as outlined in **Supplementary Figure 6a**. HEK293PAL cells were incubated under the indicated light conditions prior to measurement. eGFP expression was detected by using forward scatter height (FSC-H) *vs*. Fluorescein isothiocyanate area (FITC-A) channel (eGFP). Flow cytometry was performed using a BD FACS Canto II instrument (BD Bioscience). Data processing was performed using FlowJo (9.6.3) and GraphPad Prism (6.01).

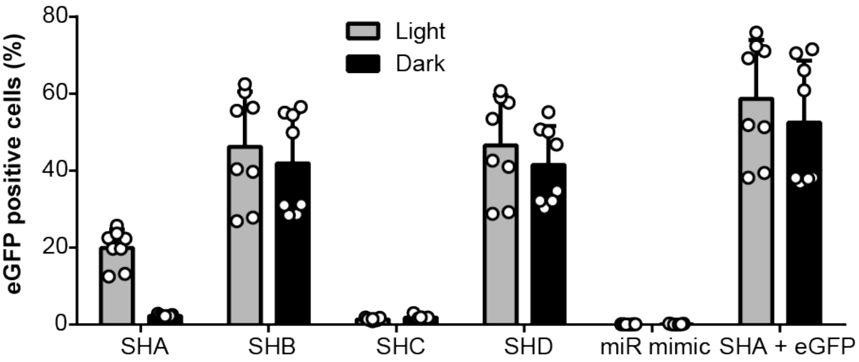

Supplementary Figure 10: Elevated levels of AGO2 increase light-control of eGFP expression mediated by a *pre*-miR21-aptamer chimera. Number of eGFP positive cells after transfection of the indicated *pre*-miR21 variants or 10 nM miR21 mimic. A normalized variant of this dataset is shown in **Fig. 2f.** eGFP expression was identified as indicated in **Supplementary Fig. 6a**. N = 8. Each biologically independent experiment was performed in duplicates. Grey bars: light conditions, black bars: dark conditions. Values are means ± s.d. Source data are provided as a source data file.

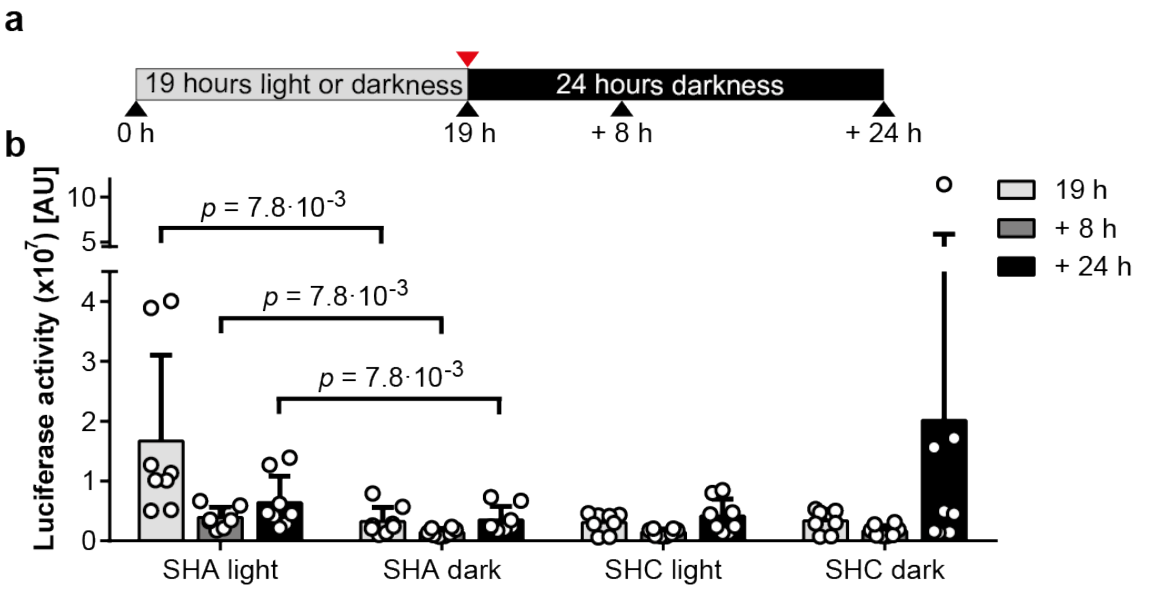

Supplementary Figure 11: Luciferase activity is reversible. a, Illumination protocol applied in (**b**). Red arrow: time point of medium exchange, black arrows, time points of sample collection. **b**, Luciferase activity after transient transfection of the indicated *pre*-miR21 variants. A normalized variant of this dataset is shown in **Fig. 2i**, in which values were normalized to aptamer point mutant *pre*-miR21 variant (SHC) incubated in darkness, where no light-dependency was expected. Normalization was performed to each time point. HEK293PAL cells were either incubated under conditions shown in (**a**) or constantly in darkness prior to measurement. **b**, Wilcoxon two-sided signed-rank test was used for statistical analysis as a paired observation was assumed. N = 8. Each biologically independent experiment was performed in duplicates. Values are means ± s.d. Source data are provided as a source data file.

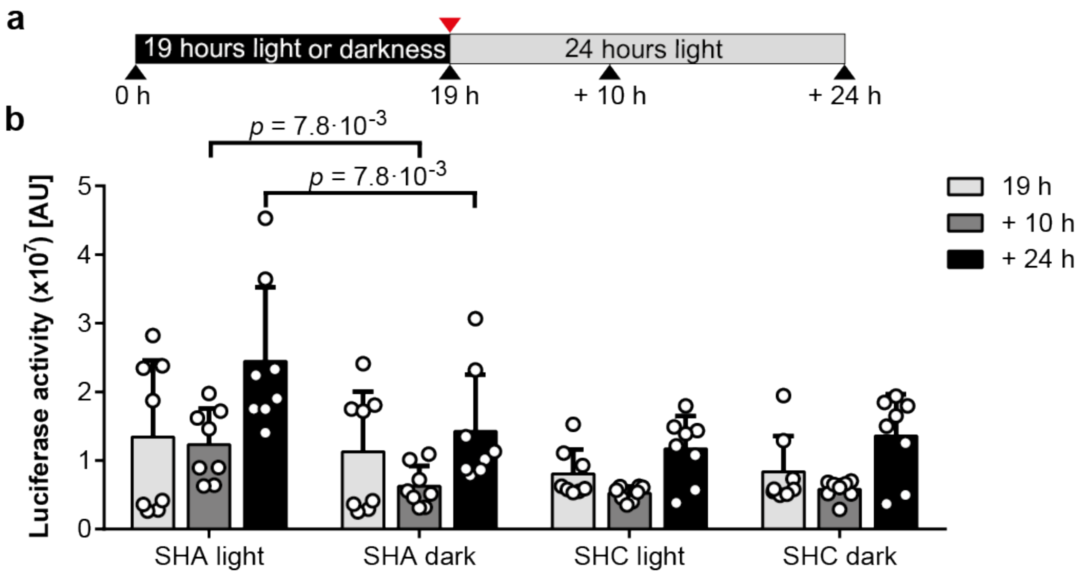

Supplementary Figure 12: Luciferase activity is inducible. a, Illumination protocol applied in (**b**). Red arrow: time point of medium exchange, black arrows, time points of sample collection. **b**, Luciferase activity after transient transfection of the indicated *pre*-miR21 variants. A normalized variant of this dataset is shown in **Fig. 2l**, in which values were normalized to aptamer point mutant *pre*-miR21 variant (SHC) incubated in darkness, where no light-dependency was expected. Normalization was performed to each time point. HEK293PAL cells were either incubated under conditions shown in (**a**) or constantly in darkness prior to measurement. **b**, Wilcoxon two-sided signed-rank test was used for statistical analysis as a paired observation was assumed. N = 8. Each biologically independent experiment was performed in duplicates. Values are means ± s.d. Source data are provided as a source data file.

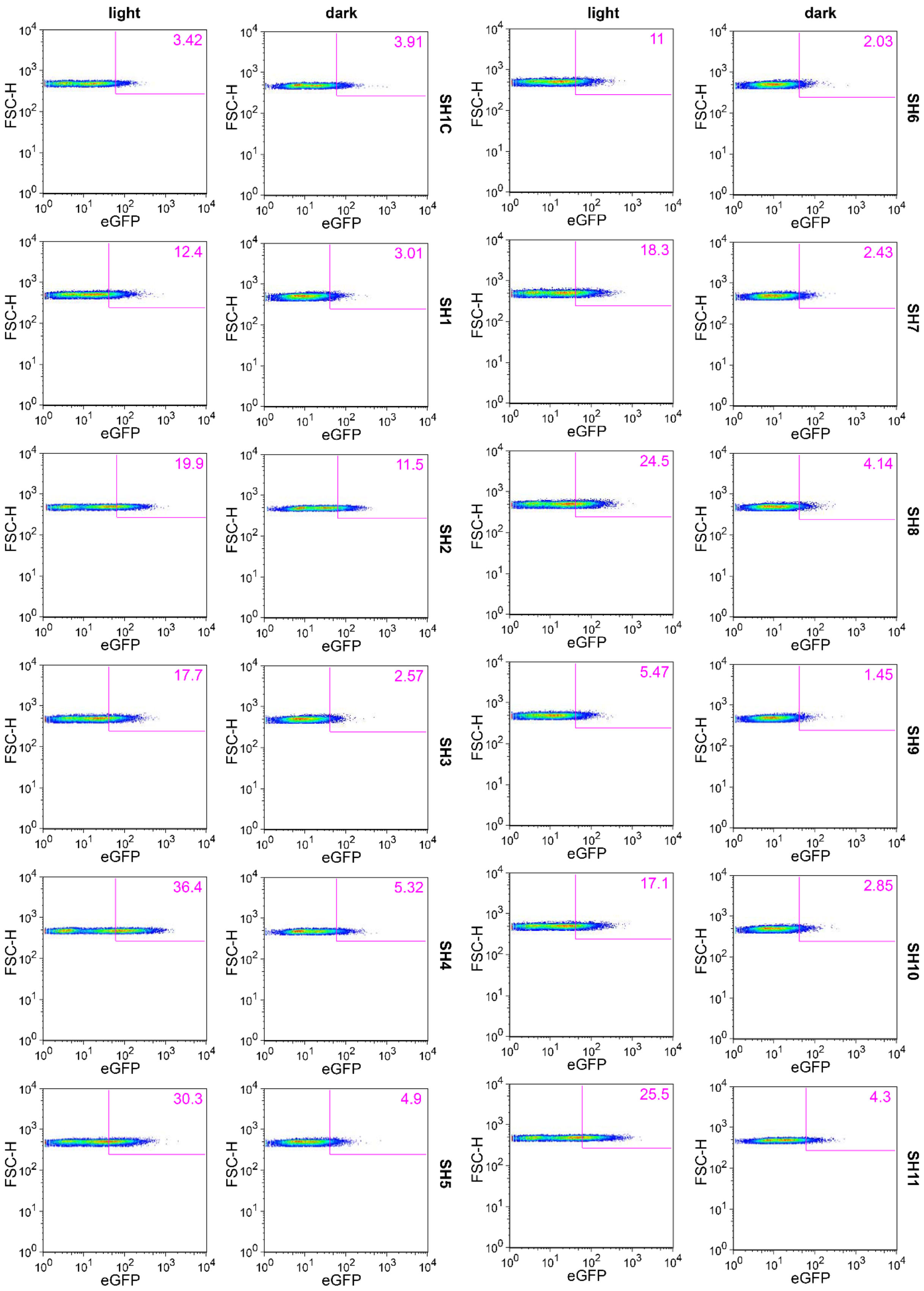
light
dark
light
dark
FSC-H
eGFP
SH1C
3.42
3.91
SH6
11
2.03
SH1
12.4
3.01
SH7
18.3
2.43
SH2
19.9
11.5
SH8
24.5
4.14
SH3
17.7
2.57
SH9
5.47
1.45
SH4
36.4
5.32
SH10
17.1
2.85
SH5
30.3
4.9
SH11
25.5
4.3

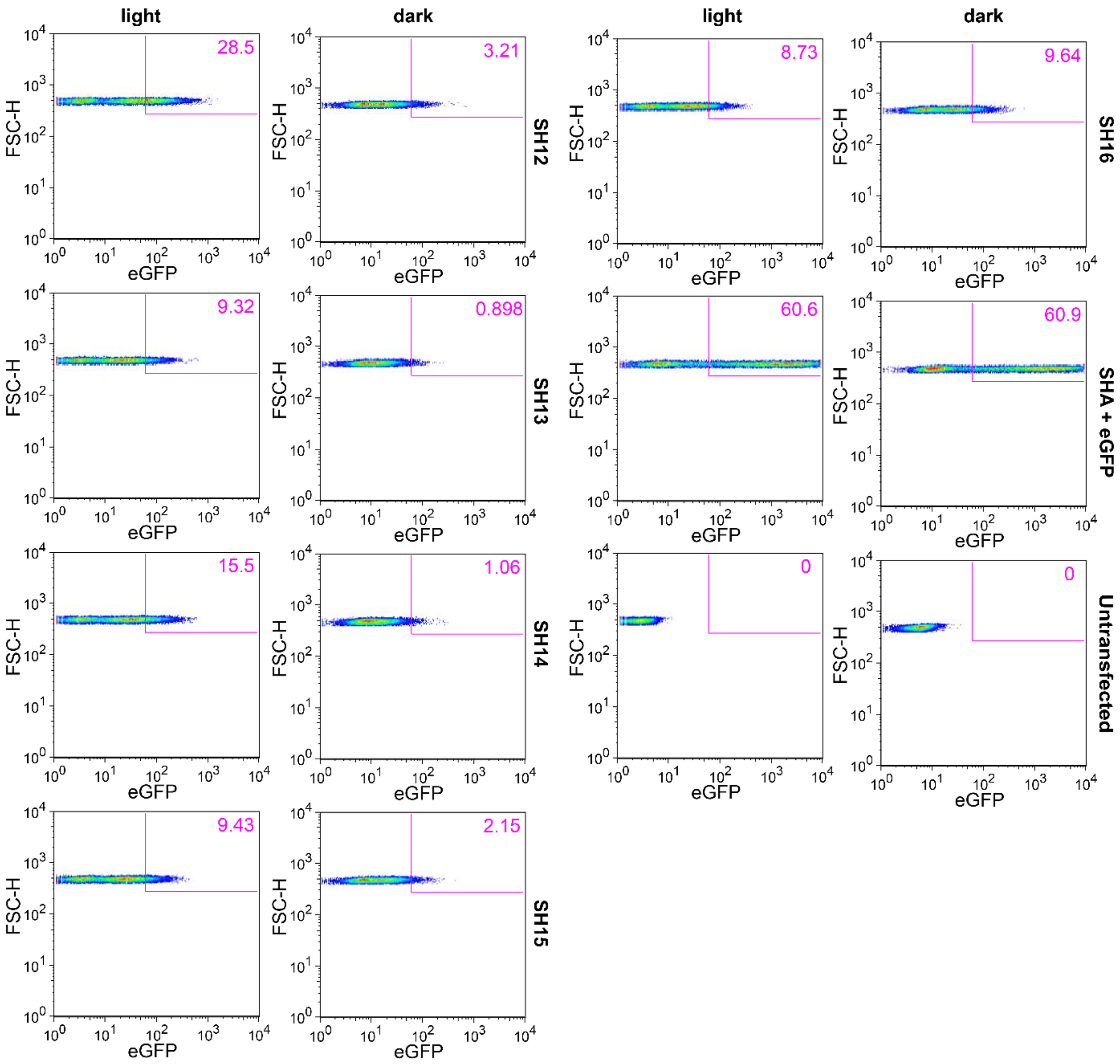

Supplementary Figure 13: Detection of eGFP expression for shRNA experiments. Representative flow cytograms of HEK293PAL cells after transfection of the indicated shRNA variants. Experiment is shown in **Fig. 3c**,**d**,**f**,**g**,**i**,**j** of the main text. Gating strategy was applied as outlined in **Supplementary Figure 6b.** HEK293PAL cells were incubated under the indicated light conditions prior to measurement. eGFP expression was detected by using forward scatter height (FSC-H) *vs*. Fluorescein isothiocyanate area (FITC-A) channel (eGFP). Flow cytometry was performed using a BD FACS Canto II instrument (BD Bioscience). Data processing was performed using FlowJo (9.6.3) and GraphPad Prism (6.01).

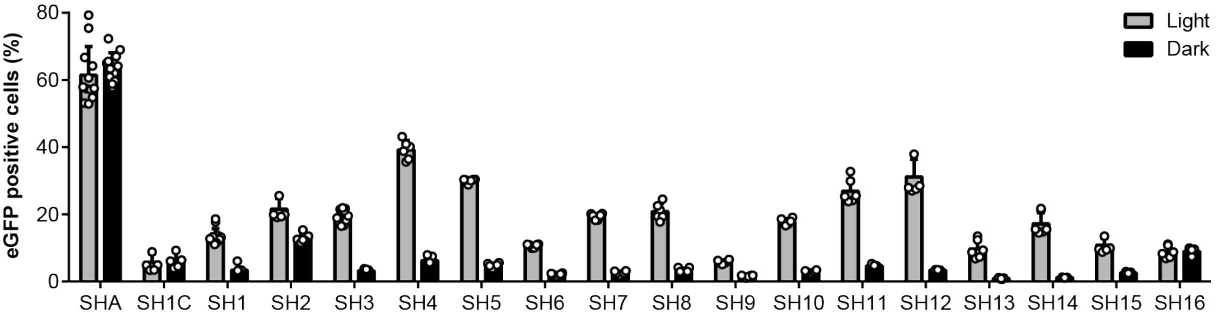

Supplementary Figure 14: shRNA-aptamer chimeras enable light-control of eGFP expression. Number of eGFP positive cells after transient transfection of AGO2 and the indicated shRNA variants. HEK293PAL cells were incubated under the indicated light conditions prior to measurement. eGFP expression was identified as indicated in **Supplementary Fig. 6a**. A normalized variant of this dataset is shown in **Fig. 3c**,**f**,**i.** N = 12 (SHA, SH1, SH3) or 6 (all others). Each biologically independent experiment was performed in duplicates. Grey bars: light conditions, black bars: dark conditions. Values are means ± s.d. Source data are provided as a source data file.

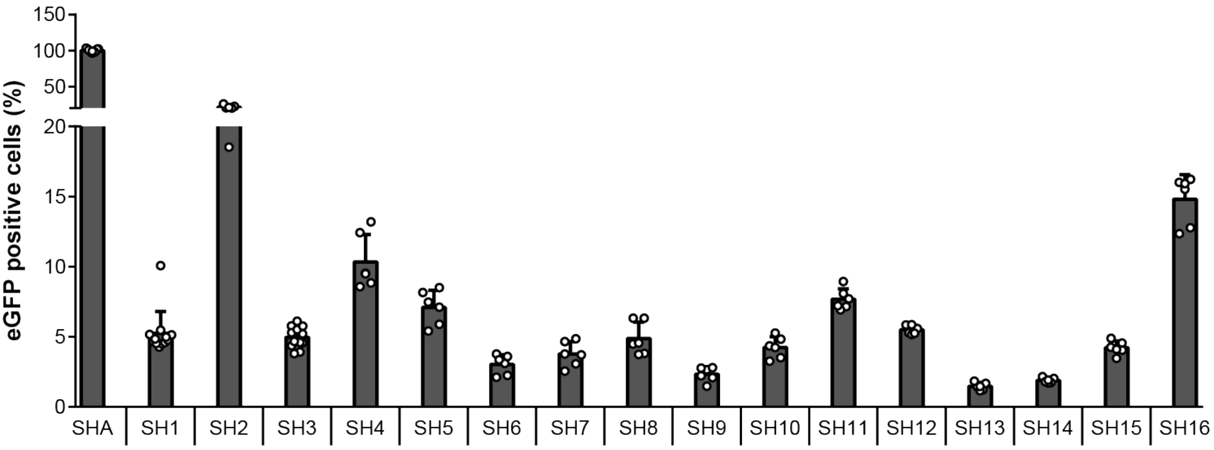

Supplementary Figure 15: HEK293PAL cells transfected with eGFP shRNAs in darkness indicate different eGFP knockdown efficiencies. Number of eGFP positive cells after transfection with the indicated shRNA. Data is also shown in **Fig. 3 c**,**f**,**i**. This figure magnifies the lower part of the y-axis to clarify eGFP knockdown differences of cells incubated in darkness. Shown are normalized values to SHA in darkness. N = 12 (SHA, SH1, SH3) or 6 (all others). Each biologically independent experiment was performed in duplicates. Dark grey bars: cells incubated in darkness. Values are means ± s.d. Source data are provided as a source data file.

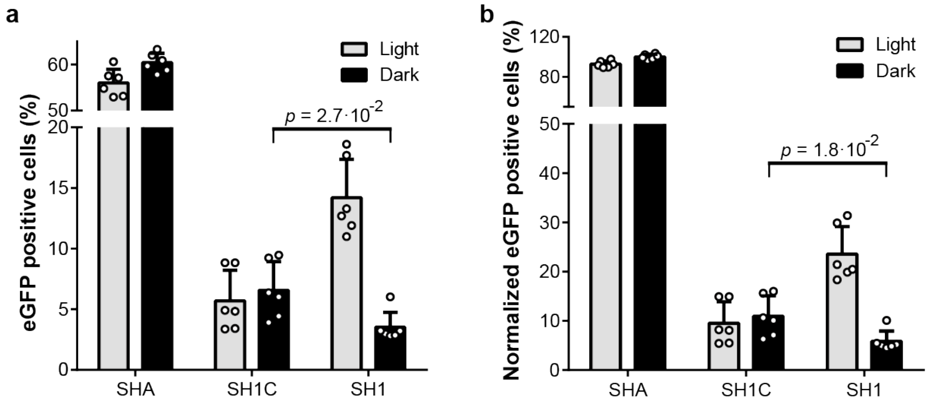

Supplementary Figure 16: Effect of the aptamer as apical loop domain on eGFP expression. a, eGFP positive cells after transfection with the indicated shRNA variants. **b**, Normalized eGFP positive cells after transfection with the indicated shRNA. **b**, Values are normalized to SHA in darkness. **a**,**b**, eGFP positive cells were identified as indicated in **Supplementary Fig. 6b**. N = 6. Each biologically independent experiment was performed in duplicates. **a**,**b**, Two-sided Mann-Whitney *U* test was used for statistical analysis as an unpaired observation was assumed. Grey bars: light conditions, black bars: dark conditions. Values are means ± s.d. Source data are provided as a source data file.

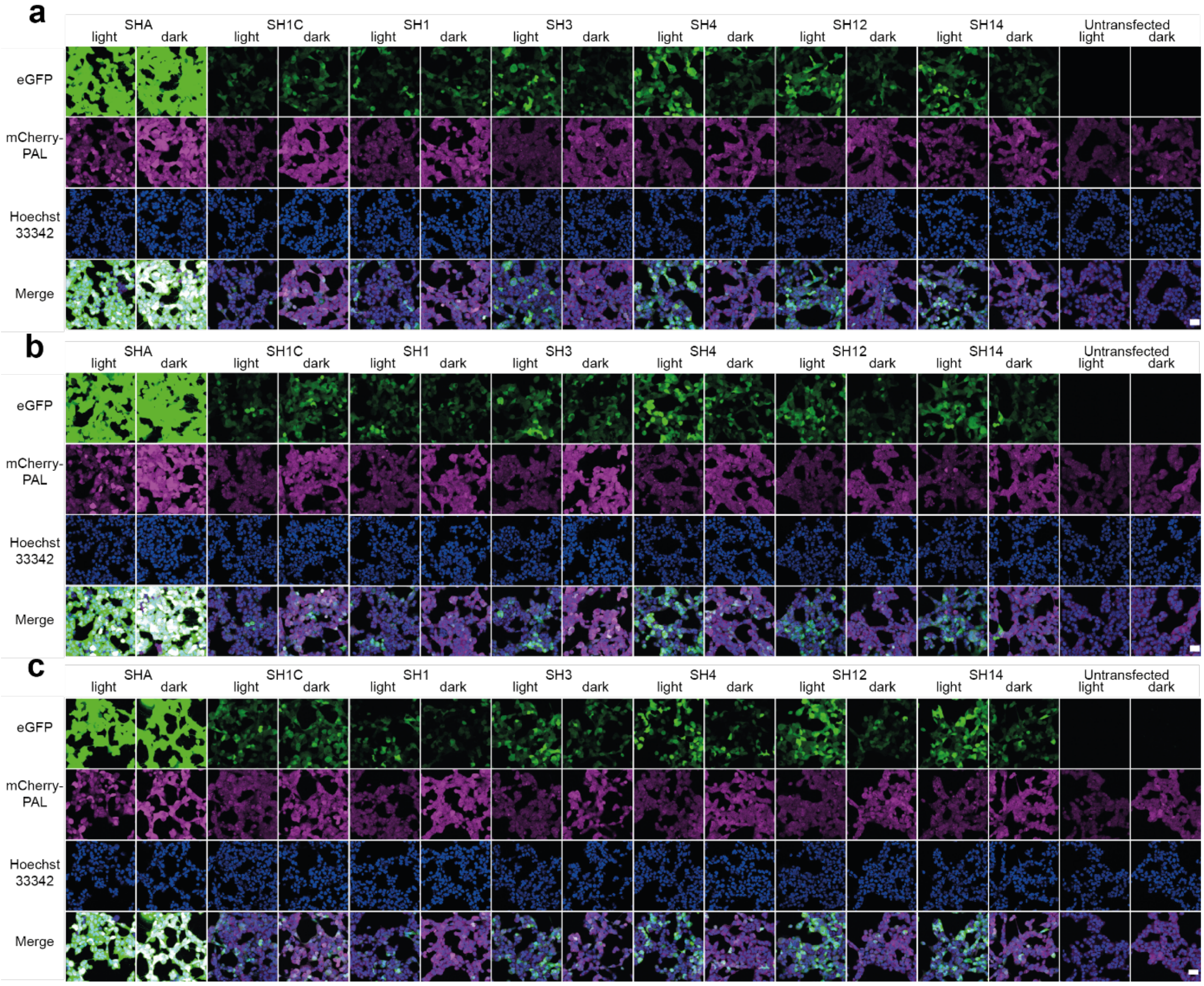

Supplementary Figure 17. Fluorescence microscopy pictures of HEK293PAL cells transfected with eGFP shRNAs indicate light-dependent eGFP expression. Fluorescence microscopy images of HEK293PAL cells transfected with the indicated shRNAs. N = 6. Each biologically independent experiment was performed in duplicates. Cells were incubated under the indicated light conditions. **a**, **b**, **c**, representatives of first, second and third repetition, respectively. Data corresponds to **Fig. 3o**, where parts of **a** are shown. Scale bar: 40 µm.

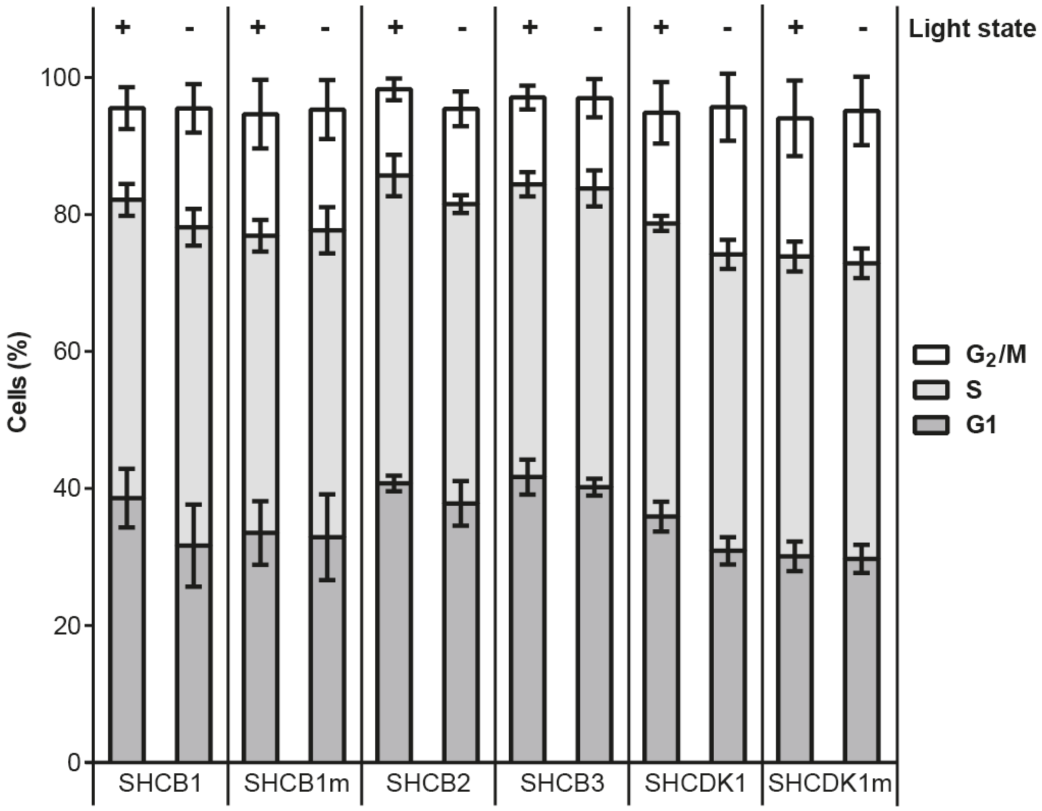

Supplementary Figure 18: Cell cycle phase distribution of cells transfected with shRNAs targeting cyclin B1 and CDK1. Percentages of HEK293PAL cells in G_1, S and G_2/M phase of cells also shown in **Fig. 4b** and **d** after transfection with the indicated shRNAs. Identity of SHCB1, SHCB1m, SHCDK1 and SHCDK1m was blinded and double-blinded in one experiment, each. N = 20 (SHCB1, SCHB1m), 6 (SHCB2, SHCB3) or 10 (SHCDK1, SHCDK1m). Each biologically independent experiment was performed in duplicates. Experiments were performed under the indicated light conditions. Values are means ± s.d. Source data are provided as a source data file.

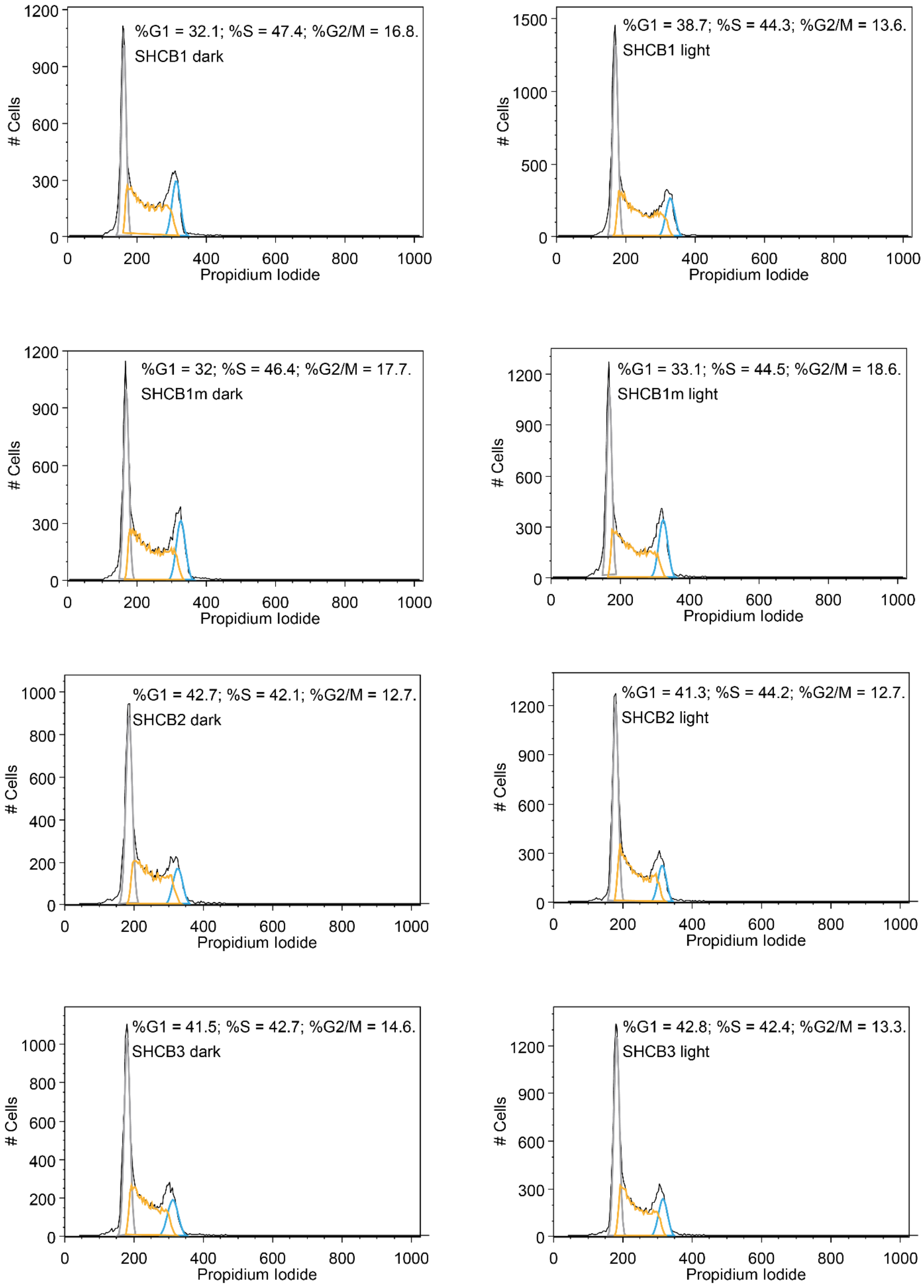
%G1 = 32.1; %S = 47.4; %G2/M = 16.8.
SHCB1 dark
%G1 = 38.7; %S = 44.3; %G2/M = 13.6.
SHCB1 light
%G1 = 32; %S = 46.4; %G2/M = 17.7.
SHCB1m dark
%G1 = 33.1; %S = 44.5; %G2/M = 18.6.
SHCB1m light
%G1 = 42.7; %S = 42.1; %G2/M = 12.7.
SHCB2 dark
%G1 = 41.3; %S = 44.2; %G2/M = 12.7.
SHCB2 light
%G1 = 41.5; %S = 42.7; %G2/M = 14.6.
SHCB3 dark
%G1 = 42.8; %S = 42.4; %G2/M = 13.3.
SHCB3 light
Cells
Propidium Iodide

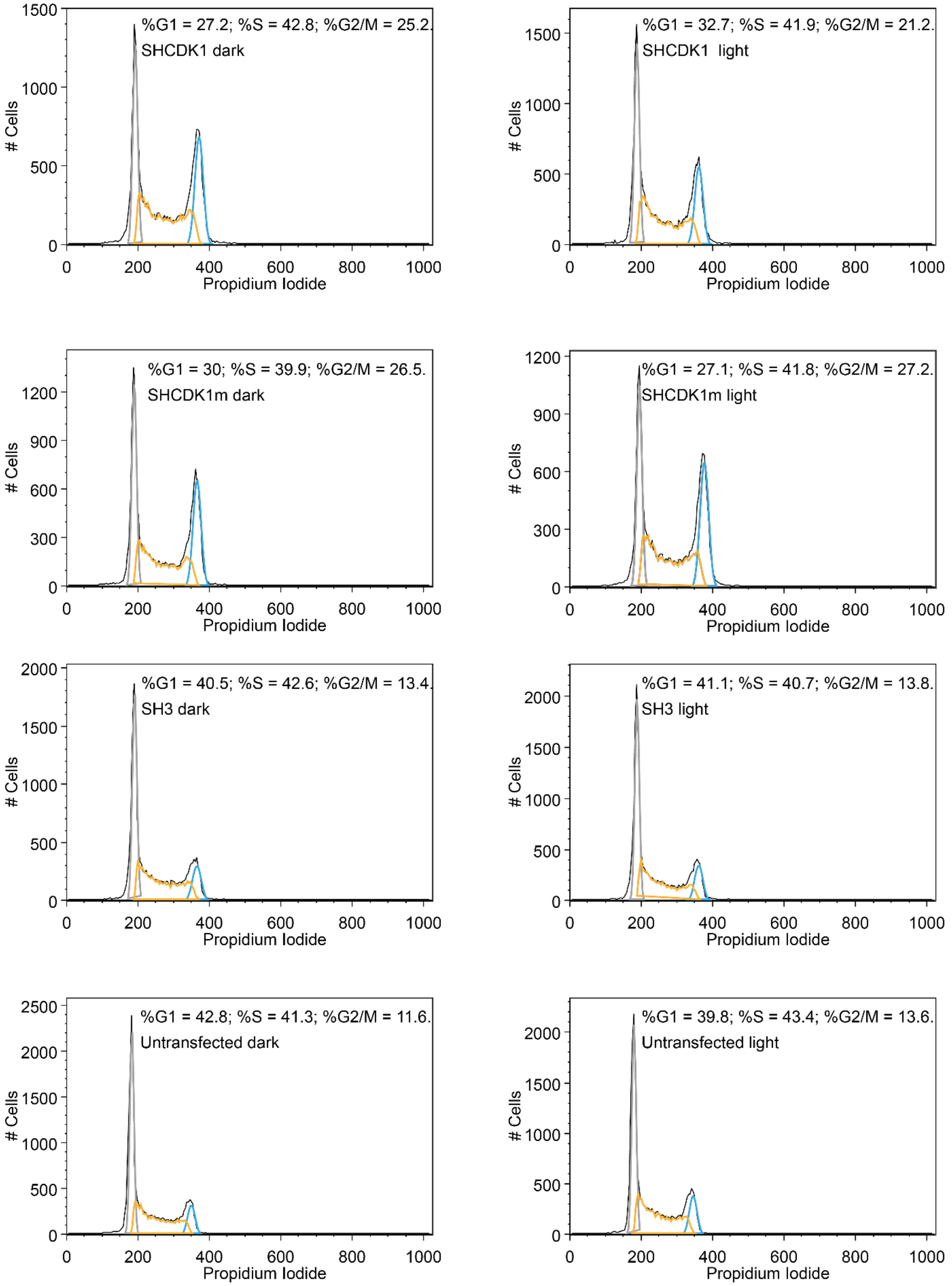

Supplementary figure 19: Cell cycle phase distribution is optoribogenetically controllable. Representative crude flow cytometry diagrams of HEK293PAL cells transfected with the indicated shRNA variants or non-treated. Cell cycle phases were detected using the cell number (# Cells) *vs.* Phycoerythrin area (depicted as Propidium Iodide). Cell cycle phase percentages were calculated using watson pragmatic algorithm. Grey: G_1 phase, orange: S phase, blue: G_2/M phase. N = at least three independent experiments performed in duplicates.

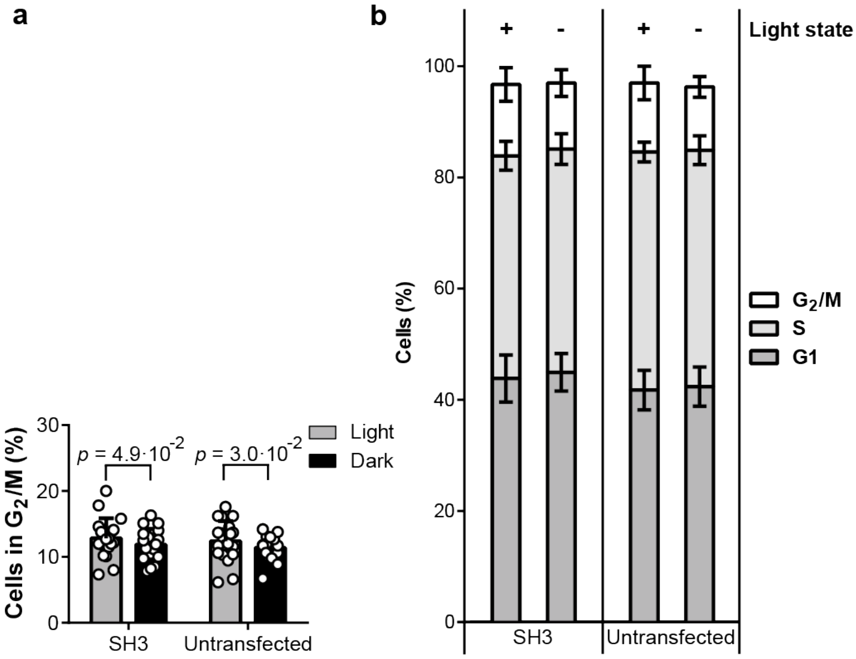

Supplementary Figure 20: Blue light slightly influences cell cycle distribution. a, Percentages of HEK293PAL cells in G_2/M phase after transfection with SH3 or no treatment. Grey bars: cells incubated under light conditions, black bars: cells incubated in darkness. **a**, Wilcoxon two-sided signed-rank test was used for statistical analysis as a paired observation was assumed. **b**, Percentages of HEK293PAL cells in G_1, S and G_2/M phase of cells shown in (**a**). **b**, Experiment was performed under the indicated light conditions. N = 20. Each biologically independent experiment was performed in duplicates. **a**,**b**, Identity of SH3 was blinded and double-blinded in one experiment, each. Values are means ± s.d. Source data are provided as a source data file.

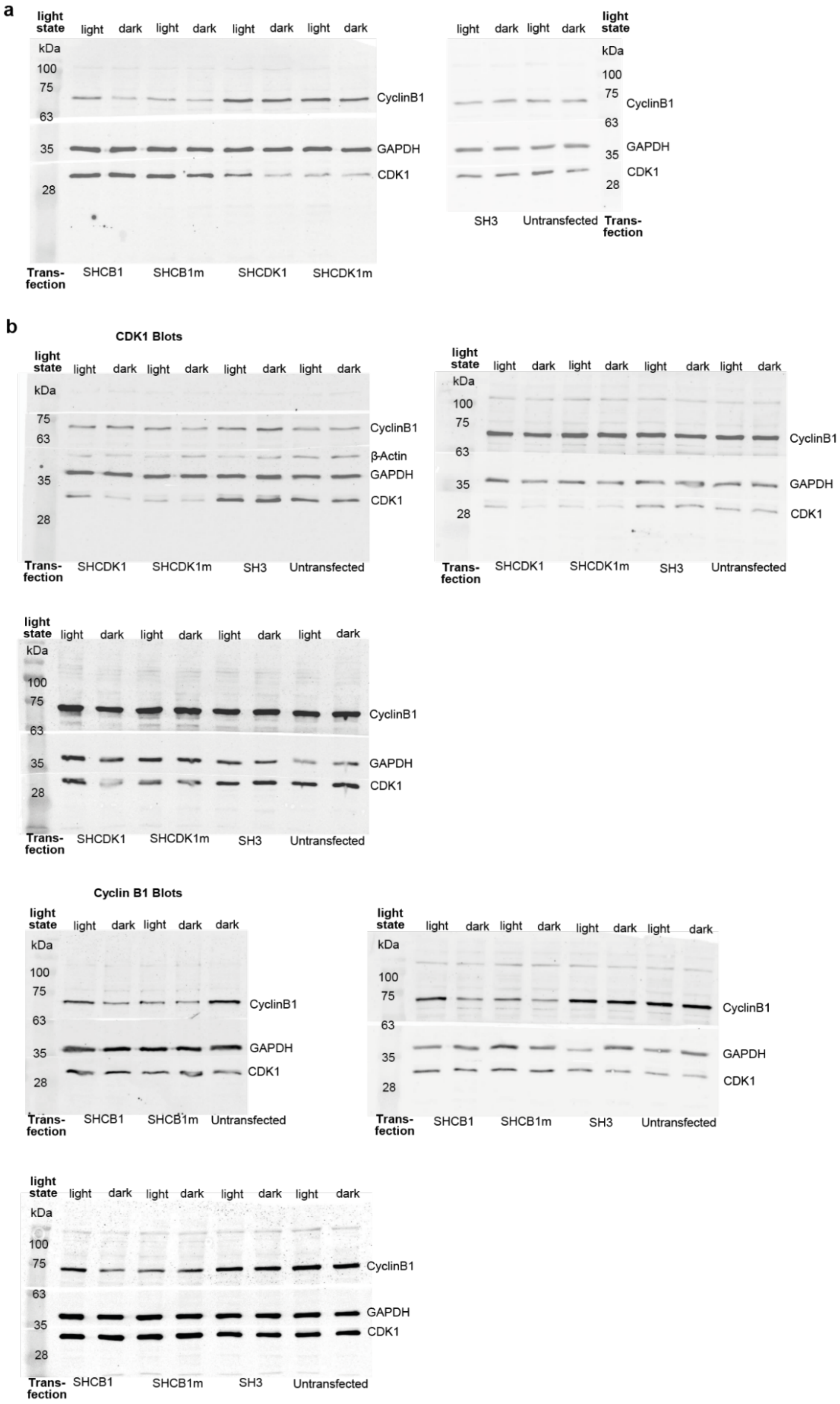

Supplementary Figure 21: Optoribogenetic control of target protein expression. Full range Western Blots showing cyclin B1, CDK1 and GAPDH protein expression after transfection with the indicated shRNAs and the indicated light state. Parts of (**a**) were shown in **fig. 4e** (**a**). Western blots shown in (**b**) were used for quantification of cyclin B1 and CDK1 protein levels using pixel densitometry shown in **Fig. 4 f,g** (**b**). N = 4. One biologically independent experiment was performed in duplicates, all others once.

Name	RNA sequence (5’-3’)
53	GGGAGGACGAUGCGGCCCGTACAGCAGCGATGCGGGTCGCGTGTCCACCCCGGCTCAGACGACUCGCU GAGGAUCCGAGA
SHA	GUAGCUUAUCAGACUGAUGUUGACGGUACAGCAGCGAUGCCGCAACACCAGUCGAUGGGCUGUUU
SHB	GAAGGCAAGCUGACCCUGAAGUUCGGUACAGCAGCGAUGCCGAAGGCAAGCUGACCCUGAAGUUU
SHC	GUAGCUUAUCAGACUGAUGUUGACGGUACAGCACCGAUGCCGCAACACCAGUCGAUGGGCUGUUU
SHD	GAAGGCAAGCUGACCCUGAAGUUCGGUACAGCACCGAUGCCGAAGGCAAGCUGACCCUGAAGUUU
SH1C	GCAAGCUGACCCUGAAGUUCA**UUCAAGAGA**UGAACUUCAGGGUCAGCUUGCUU
SH1	GCAAGCUGACCCUGAAGUUCAUCGGUACAGCAGCGAUGCCGAUGAACUUCAGGGUCAGCUUGCUU
SH2	GCACAAGCUGGAGUACAACUACGGUACAGCAGCGAUGCCGUAGUUGUACUCCAGCUUGUGCUU
SH3	GCAAGCUGACCCUGAAGUUCAUACGGUACAGCAGCGAUGCCGAUGAACUUCAGGGUCAGCUUGCUU
SH4	GCAAGCUGACCCUGAAGUUCAUUCGGUACAGCAGCGAUGCCGAUGAACUUCAGGGUCAGCUUGCUU
SH5	GCAAGCUGACCCUGAAGUUCAUGCGGUACAGCAGCGAUGCCGAUGAACUUCAGGGUCAGCUUGCUU
SH6	GCAAGCUGACCCUGAAGUUCAUCCGGUACAGCAGCGAUGCCGAUGAACUUCAGGGUCAGCUUGCUU
SH7	GCAAGCUGACCCUGAAGUUCAUCGGUACAGCAGCGAUGCCGAAUGAACUUCAGGGUCAGCUUGCUU
SH8	GCAAGCUGACCCUGAAGUUCAUCGGUACAGCAGCGAUGCCGUAUGAACUUCAGGGUCAGCUUGCUU
SH9	GCAAGCUGACCCUGAAGUUCAUCGGUACAGCAGCGAUGCCGGAUGAACUUCAGGGUCAGCUUGCUU
SH10	GCAAGCUGACCCUGAAGUUCAUCGGUACAGCAGCGAUGCCGCAUGAACUUCAGGGUCAGCUUGCUU
SH11	GCACAAGCUGGAGUACAACUAACGGUACAGCAGCGAUGCCGUAGUUGUACUCCAGCUUGUGCUU
SH12	GCACAAGCUGGAGUACAACUAUCGGUACAGCAGCGAUGCCGUAGUUGUACUCCAGCUUGUGCUU
SH13	GCACAAGCUGGAGUACAACUACGGUACAGCAGCGAUGCCGAUAGUUGUACUCCAGCUUGUGCUU
SH14	GCACAAGCUGGAGUACAACUACGGUACAGCAGCGAUGCCGUUAGUUGUACUCCAGCUUGUGCUU
SH15	GCACAAGCUGGAGUACAACUACGGUACAGCAGCGAUGCCGGUAGUUGUACUCCAGCUUGUGCUU
SH16	GCACAAGCUGGAGUACAACUACGGUACAGCACCGAUGCCGUAGUUGUACUCCAGCUUGUGCUU
SHCB1	GACACCAACUCUACAAUAUUAGUUAACGGUACAGCAGCGAUGCCGUAGCUAAUGUUGUAGAGUUGGUGU CUU
SHCB1m	GACACCAACUCUACAAUAUUAGUUAACGGUACAGCACCGAUGCCGUAGCUAAUGUUGUAGAGUUGGUGU CUU
SHCB2	GACACCAACUCUACAAUAUUAGUUAUCGGUACAGCAGCGAUGCCGUAGCUAAUGUUGUAGAGUUGGUG UCUU
SHCB3	GACACCAACUCUACAAUAUUAGUUACGGUACAGCAGCGAUGCCGUUAGCUAAUGUUGUAGAGUUGGUG UCUU
SHCDK1	GUGGAAUCUUUACAGGACUAUCACGGUACAGCAGCGAUGCCGGAUAGUCCUGUAAAGAUUCCACUU
SHCDK1 m	GUGGAAUCUUUACAGGACUAUCACGGUACAGCACCGAUGCCGGAUAGUCCUGUAAAGAUUCCACUU

Supplementary Table 1. List of *pre*-miR21 and shRNA constructs used in this study. Blue: Aptamer sequence, red: Point Mutations, orange: Hinge region nucleotides, brown (bold): control loop sequence.

Name	# Clones	Percentage (%)	RNA sequence
Mature miR21-5p	0	0	UAGCUUAUCAGACUGAUGUUGA
Mature miR21-5p – 1 nts	2	17	UAGCUUAUCAGACUGAUGUUG
Mature miR21-5p + 1 nts	2	17	UAGCUUAUCAGACUGAUGUUGA**C**
Mature miR21-5p + 2 nts	8	67	UAGCUUAUCAGACUGAUGUUGA**CG**

Supplementary Table 2. Sequencing of mature miR21-5p clones from SHA transfection indicates altered 3'-isomiR formation compared to natural *pre*-miR21. Percentage was calculated from the number of clones obtained for the respective 3'-isomiR. Blue: Nucleotides that code for the PAL-aptamer.

Primer Name	Oligonucleotide sequence (5’ to 3’)
RT-Primer	ATTCTAGAGGCCGAGGCGGCCGACATGTTTTTTTTTTTTTTTTTTTTTTTTTTTTTT
Forward Primer	CGCCTAGCTTATCAGACTGATGT
Reverse Primer	ATTCTAGAGGCCGAGGCGGCCGACATG

Supplementary Table 3. Primers used in this study.
