# Supplementary material for: Optoribogenetic control of regulatory RNA molecules
Source: Nat Commun. 2020 Sep 24;11:4825. doi: 10.1038/s41467-020-18673-5 (PMC7518282; doi:10.1038/s41467-020-18673-5)
Supplement: Supplementary file 2 — Reporting Summary [file 41467_2020_18673_MOESM2_ESM.pdf]

nature research

Corresponding author(s): Günter Mayer

Last updated by author(s): Aug 26, 2020

Reporting Summary

Nature Research wishes to improve the reproducibility of the work that we publish. This form provides structure for consistency and transparency in reporting. For further information on Nature Research policies, see our Editorial Policies and the Editorial Policy Checklist.

Statistics

For all statistical analyses, confirm that the following items are present in the figure legend, table legend, main text, or Methods section.

n/a	Confirmed	
☐	☒	The exact sample size (n) for each experimental group/condition, given as a discrete number and unit of measurement
☐	☒	A statement on whether measurements were taken from distinct samples or whether the same sample was measured repeatedly
☐	☒	The statistical test(s) used AND whether they are one- or two-sided<br>*Only common tests should be described solely by name; describe more complex techniques in the Methods section.*
☒	☐	A description of all covariates tested
☒	☐	A description of any assumptions or corrections, such as tests of normality and adjustment for multiple comparisons
☐	☒	A full description of the statistical parameters including central tendency (e.g. means) or other basic estimates (e.g. regression coefficient) AND variation (e.g. standard deviation) or associated estimates of uncertainty (e.g. confidence intervals)
☐	☒	For null hypothesis testing, the test statistic (e.g. F, t, r) with confidence intervals, effect sizes, degrees of freedom and P value noted<br>*Give P values as exact values whenever suitable.*
☒	☐	For Bayesian analysis, information on the choice of priors and Markov chain Monte Carlo settings
☒	☐	For hierarchical and complex designs, identification of the appropriate level for tests and full reporting of outcomes
☐	☒	Estimates of effect sizes (e.g. Cohen's d, Pearson's r), indicating how they were calculated

Our web collection on statistics for biologists contains articles on many of the points above.

Software and code

Policy information about availability of computer code

Data collection	Luciferase data was acquired using EnSpire Multilabel Reader software version 3.0 (PerkinElmer);<br>Flow cytometry data was acquired using FACSDiva v.6.1.3 (BD Biosciences);<br>Fluorescence microscopy images were aquired using Zen 2012 (Zeiss);<br>Western Blots were collected with Oyssey Application Software Version 2.1.12 (Li-cor);<br>In vitro binding data was collected with Xfluor4 Version: V 4.51 (Tecan)
Data analysis	Data was analyzed using GraphPad Prism 6.01 (Graphpad Software Inc.);<br>Flow cytometry data was analyzed using FlowJo 9.6.3 (BD Biosciences);<br>Western Blots were analyzed using Fiji software package (ImageJ 1.52p)

For manuscripts utilizing custom algorithms or software that are central to the research but not yet described in published literature, software must be made available to editors and reviewers. We strongly encourage code deposition in a community repository (e.g. GitHub). See the Nature Research guidelines for submitting code & software for further information.

Data

Policy information about availability of data

All manuscripts must include a data availability statement. This statement should provide the following information, where applicable:

- Accession codes, unique identifiers, or web links for publicly available datasets
- A list of figures that have associated raw data
- A description of any restrictions on data availability

Source data are provided with this paper. All other data are available in the main text, the Supplementary Information, or from the corresponding author upon request. Plasmids from Addgene (#10822 [https://www.addgene.org/10822]) were used in this study.

Field-specific reporting

Please select the one below that is the best fit for your research. If you are not sure, read the appropriate sections before making your selection.

- [x] Life sciences
- [ ] Behavioural & social sciences
- [ ] Ecological, evolutionary & environmental sciences

For a reference copy of the document with all sections, see nature.com/documents/nr-reporting-summary-flat.pdf

Life sciences study design

All studies must disclose on these points even when the disclosure is negative.

Sample size	No sample-size calculation was performed. For all measurements, at least three biological replicates (i.e. independent samples) were analyzed as this is the standard in the field. In each biological replicate, two technical replicates were included. Based on the resultant standard deviation across measurements, sample sizes were considered as appropriate. Cellular responses were sufficiently robust to observe clear results for light-induced effects which set them apart from the controls.
Data exclusions	No data were excluded from the analysis. Some samples for technical replicates depicted in Supplementary Fig. 20 could not be evaluated as the Watson pragmatic algorithm could not be applied to these samples by the software (FlowJo 9.6.3).
Replication	All experiments were repeated in a minimum of 3 biological replicates to ensure reproducibility and all attempts at reproduction were successful. All replications yielded similar results; no replication failed. The Photomask experiment was performed once.
Randomization	Not relevant, because the study did not involve experiments on animals or humans. All experiments were conducted with purified components, in bacterial culture or in mammalian cell culture.
Blinding	The identity of SHCB1, SHCB1m, SHCDK1, SHCDK1m and SH3 which are shown in Fig. 3b, d and Supporting Figure 7 and 9 were blinded and double-blinded in one experiment, each. Since all cell cultures, treatments and measurements were performed under identical conditons, no further blinding was applied.

Reporting for specific materials, systems and methods

We require information from authors about some types of materials, experimental systems and methods used in many studies. Here, indicate whether each material, system or method listed is relevant to your study. If you are not sure if a list item applies to your research, read the appropriate section before selecting a response.

Materials & experimental systems

n/a	Involved in the study	
[ ]	[x]	Antibodies
[ ]	[x]	Eukaryotic cell lines
[x]	[ ]	Palaeontology and archaeology
[x]	[ ]	Animals and other organisms
[x]	[ ]	Human research participants
[x]	[ ]	Clinical data
[x]	[ ]	Dual use research of concern

Methods

n/a	Involved in the study	
[x]	[ ]	ChIP-seq
[ ]	[x]	Flow cytometry
[x]	[ ]	MRI-based neuroimaging

Antibodies

Antibodies used	1. CDK1: mouse anti-cdc2, Cell Signaling, POH1, #9116;<br>2. GAPDH: mouse-anti-GAPDH, Santa Cruz Biotechnology, sc-47724;<br>3. Cyclin B1: goat anti-Cyclin B1, R&D Systems, AF6000;<br>4. IRDye 800CW goat anti-mouse (Li-cor 926-32210);<br>5. IRDye 800CW donkey anti-goat (Li-cor 926-32214);

6. IRDye 800CW goat anti-mouse (Li-cor 926-32211).

Validation	Validation of the antibodies for Western blotting was performed by the vendors. In detail:<br>1.: cdc2 (POH1) Mouse mAb detects endogenous levels of total cdc2 protein in HeLa cells synchronized at various stages of the cell cycle (e.g. G0, G1S, G2 and M). The antibody does not cross-react with other cdks. Species Reactivity: Human, Monkey<br>2.: Reacts with GAPDH from A549, HeLa and Hep G2 cells (human origin). Does not react with mouse and rat GAPDH.<br>3.: Reacts with Cyclin B1 from U2OS, K562, HeLa, Jurkat (human origin). Also reacts with mouse Cyclin B1.<br>In addition, bands were identified at expected molecular weights for protein products.

Eukaryotic cell lines

Policy information about cell lines

Cell line source(s)	1. HEK293 cells, purchased from CLS Cell Lines Service GmbH, Eppelheim, Germany<br>2. HEK293PAL cell line was generated from the above-mentioned cell line (1.) and is availible from the corresponding author upon request
Authentication	The identity of the HEK293 cells was verified by the vendor via STR DNA profiling.
Mycoplasma contamination	The absence of Mycoplasma contamination was confirmed by the vendor and tested every three months using PCR detection. For the positive control reaction, the mycoplasma DNA amplicon (265 - 278 bp) could be detected. For the negative control reaction, the internal control band (191 bp) could be detected. For reactions containing HEK293 or HEK293PAL supernatant, the internal control band (191 bp) could be detected.
Commonly misidentified lines (See ICLAC register)	None used in this study.

Flow Cytometry

Plots

Confirm that:

- [x] The axis labels state the marker and fluorochrome used (e.g. CD4-FITC).
- [x] The axis scales are clearly visible. Include numbers along axes only for bottom left plot of group (a 'group' is an analysis of identical markers).
- [x] All plots are contour plots with outliers or pseudocolor plots.
- [x] A numerical value for number of cells or percentage (with statistics) is provided.

Methodology

Sample preparation	Not relevant, because the study did not involve experiments on animals or humans. All experiments were conducted with purified components, in bacterial culture or in mammalian cell culture.
Instrument	BD FACSCanto II, BD Biosciences
Software	FloJo version 9.6.3
Cell population abundance	Not relevant, because the study did not involve experiments on animals or humans. All experiments were conducted with purified components, in bacterial culture or in mammalian cell culture.
Gating strategy	Gating strategies are described in detail in Supporting Figure 6

- [x] Tick this box to confirm that a figure exemplifying the gating strategy is provided in the Supplementary Information.
